# Lipidomic Analysis Reveals Serum Alteration of Plasmalogens in Patients Infected With ZIKA Virus

**DOI:** 10.3389/fmicb.2019.00753

**Published:** 2019-04-12

**Authors:** Adriano Queiroz, Isabella Fernanda Dantas Pinto, Maricélia Lima, Marta Giovanetti, Jaqueline Goes de Jesus, Joilson Xavier, Fernanda Khouri Barreto, Gisele André Baptista Canuto, Helineide Ramos do Amaral, Ana Maria Bispo de Filippis, Denise Lima Mascarenhas, Melissa Barreto Falcão, Normeide Pedreira Santos, Vasco Ariston de Carvalho Azevedo, Marcos Yukio Yoshinaga, Sayuri Miyamoto, Luiz Carlos Junior Alcantara

**Affiliations:** ^1^Laboratório de Patologia Experimental, Instituto Gonçalo Moniz, Salvador, Brazil; ^2^Departamento de Bioquímica, Instituto de Química, Universidade de São Paulo, São Paulo, Brazil; ^3^Departamento de Saúde, Universidade Estadual de Feira de Santana, Feira de Santana, Brazil; ^4^Laboratório de Flavivírus, Instituto Oswaldo Cruz, Rio de Janeiro, Brazil; ^5^Instituto Multidisciplinar em Saúde, Universidade Federal da Bahia, Vitória da Conquista, Brazil; ^6^Departamento de Química Analítica, Instituto de Química, Universidade Federal da Bahia, Salvador, Brazil; ^7^Núcleo de Pesquisa em Vigilância da Saúde, Universidade Estadual de Feira de Santana, Feira de Santana, Brazil; ^8^Secretaria Municipal de Saúde do Município de Feira de Santana, Feira de Santana, Brazil; ^9^Instituto de Ciências Biológicas, Universidade Federal de Minas Gerais, Belo Horizonte, Brazil; ^10^Laboratório de Genética Celular e Molecular, Instituto de Ciências Biológicas, Universidade Federal de Minas Gerais, Belo Horizonte, Brazil

**Keywords:** Zika virus, serum lipidomics, plasmalogens, phosphatidylethanolamine, arbovirus

## Abstract

Zika virus (ZIKV) is an arthropod-borne virus (arbovirus) in the *Flavivirus* genus of the *Flaviviridae* family. Since the large outbreaks in French Polynesia in 2013–2014 and in Brazil in 2015, ZIKV has been considered a new public health threat. Similar to other related flavivirus, ZIKV is associated with mild and self-limiting symptoms such as rash, pruritus, prostration, headache, arthralgia, myalgia, conjunctivitis, lower back pain and, when present, a short-term low grade fever. In addition, ZIKV has been implicated in neurological complications such as neonatal microcephaly and Guillain–Barré syndrome in adults. Herein, serum lipidomic analysis was used to identify possible alterations in lipid metabolism triggered by ZIKV infection. Patients who presented virus-like symptoms such as fever, arthralgia, headache, exanthema, myalgia and pruritus were selected as the control group. Our study reveals increased levels of several phosphatidylethanolamine (PE) lipid species in the serum of ZIKV patients, the majority of them plasmenyl-phosphatidylethanolamine (pPE) (or plasmalogens) linked to polyunsaturated fatty acids. Constituting up to 20% of total phospholipids in humans, plasmalogens linked to polyunsaturated fatty acids are particularly enriched in neural membranes of the brain. The biosynthesis of plasmalogens requires functional peroxisomes, which are important sites for viral replication, including ZIKV. Thus, increased levels of plasmalogens in serum of ZIKV infected subjects suggest a link between ZIKV life cycle and peroxisomes. Our data provide important insights into specific host cellular lipids that are likely associated with ZIKV replication and may serve as platform for antiviral strategy against ZIKV.

## Introduction

The ZIKA virus (ZIKV) is an arbovirus that contains a single-stranded RNA genome. Like other *Flavivirus*, its nucleic acid encodes a polyprotein that is processed into three viral particle structural proteins, namely the capsid, the precursor of membrane, and the envelope proteins. Also composing the ZIKV structure are seven non-structural proteins NS1 to NS5 and a lipid bilayer envelope (Kuno et al., 1998; Dai et al., 2016). The illnesses caused by ZIKV may be either asymptomatic or may produce mild symptoms such as fever, skin rash, joint pain, myalgia and conjunctivitis (Malone et al., 2016), resembling the symptoms of dengue and chikungunya. ZIKV can also induce severe diseases such as neonatal microcephaly and Guillain–Barré syndrome in adults (White et al., 2016).

The replication of flaviviruses is fully dependent on host cell machinery and all steps in the flavivirus’ lifecycle are closely connected with either cellular or viral lipids (Mukhopadhyay et al., 2005; Martin-Acebes et al., 2016). For example, the attachment of West Nile and dengue viruses is dependent on specific host cell receptors that are recognized by the viral envelope components phosphatidylserine (PS) and phosphatidylethanolamine (Richard et al., 2015; Carnec et al., 2016). Also, for cell entry and fusion with endocytic compartments, flavivirus such as the Yellow Fever and the Japanese Encephalitis-virus-like particles modulate phosphatidylinositol-3-phosphate signaling and alter membrane phospholipid distribution (Nour et al., 2013). And finally, virus replication and assembly only occur after a proper flavivirus-induced rearrangement of membrane structures (Apte-Sengupta et al., 2014; Harak and Lohmann, 2015). West Nile and dengue viruses’ proteins co-localize with the endoplasmic reticulum and Golgi complex and induce alterations in their membranes (Roosendaal et al., 2006; Miller et al., 2007; Kaufusi et al., 2014). More recently, ZIKV has been shown to target several host cell organelles involved in lipid biosynthesis and metabolism, including the endoplasmic reticulum, peroxisome and lipid droplets (Coyaud et al., 2018).

Most of the experiments evidencing the importance of host cell lipids for flavivirus’ lifecycle such as envelope biogenesis and replication were conducted *in vitro*. However, few studies have detected significant lipidome alterations induced by Hepatitis B virus (HBV), dengue virus and ZIKV in the serum of infected patients (Cui et al., 2013; Schoeman et al., 2016; Melo et al., 2017, 2018). For example, important changes in serum lipids (e.g., phospholipids and sphingolipids) as well as alterations in lipid-related metabolic pathways (e.g., fatty acid biosynthesis and β-oxidation, phospholipid catabolism) were detected in patients during the temporal progression of DENV infection (Cui et al., 2013). In a metabolomics study, Melo et al. (2017) observed alterations in the ganglioside GM2, a glycosphingolipid, and different species of phosphatidylinositol phosphates in the serum of ZIKV-infected patients. Similar to the above-mentioned studies, we applied an untargeted lipidomic analysis of serum using liquid chromatography coupled to mass spectrometry to identify possible lipid alterations in ZIKV infected patients. In line with recent findings on the interaction of flavivirus with host cell peroxisomes (Tanner et al., 2014; You et al., 2015; Coyaud et al., 2018), we highlight here alterations in serum plasmalogens levels that are likely associated with ZIKV infection.

## Materials and Methods

### Study Participants

A total of 24 participants with symptoms corresponding to arbovirus infection were enrolled in this study and divided into ZIKV and control groups. This distinction was based on a diagnostic test for Zika by real-time reverse transcription polymerase chain reaction (RT-qPCR). Probe-based RT-qPCR against the prM target, using the following set of primers: CCGCTGCCCAACACAAG (forward), CCACTAACGTTCTTTTGCAGACAT (reverse), and FAM AGCCTACCTTGACAAGCAGTCAGACACTCAA as the probe reporter dye, was performed as previously described (Lanciotti et al., 2008). The specificity of the ZIKV primers was previously evaluated and no reactivity with DENV-1, DENV-2, DENV-3, DENV-4, WNV, St. Louis encephalitis virus, YFV, Powassan virus, Semliki Forest virus, o’nyong-nyong virus, chikungunya virus, and Spondweni virus (SPOV) were observed (Lanciotti et al., 2008). Primers and probes used in the diagnostic assay were targeting the Asian genotype, which is currently circulating in Brazil and in the Americas (Faria et al., 2017). The ZIKV group was composed of 12 individuals and the 12 participants belonging to control group were symptomatic but tested negative when submitted to RT-qPCR. The samples were collected from May 2015 to November 2016 and all subjects were residents of Feira de Santana, a city located in the state of Bahia (Northeast Brazil). All participants were negative for chikungunya infection based on a diagnostic RT-qPCR test, which was performed as previously described (Lanciotti et al., 2007). The institutional review boards at the Instituto Gonçalo Moniz (Fiocruz-Bahia) approved the present study (CAAE: 1.100.349) and the subjects provided written and informed consent prior to participation.

### Chemicals and Reagents

The following lipids used as internal standards were purchased from Avanti Polar Lipids, Inc., (Alabaster, AL, United States): 1-heptadecanoyl-2-hydroxy-sn-glycero-3-phosphocoline (LPC 17:0), 1,2-diheptadecanoyl-sn-glycero-3-phosphocholine (PC 17:0/17:0), 1,2-diheptadecanoyl-sn-glycero-3-phosphoet -hanolamine (PE 17:0/17:0) and N-heptadecanoyl-D-erytro-sp -hingosylphosphorylcholine (SM d18:1/17:0), N-heptadecanoyl-D-erytro-sphingosine (Cer d18:1/17:0). Other internal standards and reagents such as 1,2,3-tritetradecanoylglycerol (TG 14:0/14:0/14:0), methyl tert-butyl ether (MTBE), ammonium formate and ammonium acetate were obtained from Sigma-Aldrich (St. Louis, MO, United States). All organic solvents were of high-performance liquid chromatography (HPLC) grade obtained from Sigma-Aldrich (St. Louis, MO, United States).

### Lipid Extraction

Serum samples (50 μL) from 12 control and 12 ZIKV-infected patients were extracted by a modified MTBE method (Matyash et al., 2008). Briefly, serum samples were mixed with 50 μL of internal standards (10 μg/mL) and 200 μL of ice-cold methanol. After thoroughly vortexing for 10 s, 1 mL of MTBE was added to the mixture, which was stirred for 1 h at 20°C. Next, 300 μL of water was added to the mixture, followed by vortexing 10 s and resting in an ice bath for 10 min. After centrifugation at 10,000 × *g* for 10 min at 4°C, the supernatant containing the lipid extract was transferred to a vial and dried under N_2_ gas. The extracted lipids were re-dissolved in 100 μL of isopropanol and centrifuged at 1500 × *g* for 3 min at 4°C before liquid chromatography coupled to high-resolution mass spectrometry (LC-MS) analysis.

### LC-MS Analysis

Lipid extracts were analyzed in an untargeted method using ultra-high performance liquid chromatography (Nexera UHPLC, Shimadzu, Kyoto, Japan) coupled to a TripleTOF6600 mass spectrometer (Sciex, Concord, United States) with electrospray ionization in both negative and positive modes. Lipid extracts were loaded into a CORTECS^®^ column (UPLC C18 column, 1.6 μm, 2.1 mm i.d. 100 mm). The mobile phases comprised of (A) water/acetonitrile (60:40) and (B) isopropanol/acetonitrile/water (88:10:2) both with 10 mM ammonium acetate or ammonium formate for analysis in negative or positive mode, respectively. The gradient was started from 40 to 100% over the first 10 min, hold at 100%B from 10 to 12 min, decreased from 100 to 40% B during 12–13 min, and hold at 40% B from 13 to 20 min. The flow rate was 0.2 mL/min and column temperature was maintained at 35°C. The sample injection volume was 1 μL.

The MS operated in Information Dependent Acquisition (IDA^®^) acquisition mode with scan range set a mass-to-charge ratio of 100–2000 Da. Data were obtained in a period cycle time of 1.05 s with 100 ms acquisition time for MS1 scan and 25 ms acquisition time to obtain MS/MS of the top 36 precursor ions. Data acquisition was performed using Analyst^®^ 1.7.1 with an ion spray voltage of -4.5 kV and 5.5 kV for negative and positive modes, respectively, and the cone voltage at ± 80 V. The curtain gas was set at 25 psi, nebulizer and heater gases at 45 psi and interface heater of 450°C.

### Data Processing

The most abundant precursor ions detected by the IDA, covering 70–80% of total ion counts, were individually identified by inspection of their MS/MS using PeakView^®^ (Sciex, Concord, United States). The ESI negative mode was the method of choice for the identification of free fatty acids, glycerophospholipids and sphingolipids, while the ESI positive mode was used to identify neutral lipids such as triglycerides and cholesteryl esters. For quantification, the peak area of each lipid molecular species was obtained from its precursor ion and normalized by the peak area of the corresponding internal standard (Supplementary Table S1) using MultiQuant^®^ (Sciex, Concord, United States). Lipid concentrations were calculated based on the ratio between integrated MS data of the lipid species and the serum volume used for lipid extraction. Lipid concentrations were expressed as μg of lipid per μl of serum.

### Statistical Analysis

Multivariate and univariate analyses were carried out using the online software Metaboanalyst 4.0 (Chong et al., 2018), and all the data were subjected to log transformation prior to analysis. Sparse partial least squares discriminant analysis (sPLS-DA) was used to obtain an overall picture of the data set. A volcano plot analysis to identify altered lipid profiles was performed with fold change (FC) > 1.5 and *p*-value < 0.05 by Student’s *t*-test. Receiver operating characteristic (ROC) curve analysis was constructed and the area under ROC curves (AUC) was calculated to investigate whether the characteristics of the lipid that differed significantly between two groups could be efficiently exploited for constructing a sensitive biomarker of ZIKV infection.

## Results

### Subjects’ Clinical and Demographic Characteristics

Table 1 shows the clinical and demographic characteristics of 24 patients participating in this study, which included ZIKV infected (*n* = 12) and control (*n* = 12) groups. While the number of subjects used in our study is slightly lower than published investigations with flavivirus infected patients (Cui et al., 2013; Schoeman et al., 2016; Melo et al., 2017, 2018), pronounced differences in metabolic and lipid profiles of serum were reported for ZIKV (*n* = 35) and control groups (*n* = 44) (Melo et al., 2017). In order to identify the lipid molecular species specifically altered by ZIKV infection, both ZIKV and control groups were symptomatic. More than 75% of the participants in both groups presented arthralgia, headache, exanthema, pruritus and prostration. Tingling and dormancy were observed in 58% of the ZIKV subjects against 25% in the control group. Among the clinical characteristics, only retro-orbital pain (ZIKV = 92% and control = 17%) and fever (ZIKV = 92% and control = 50%) were more frequently observed in ZIKV patients than controls (Pearson’s chi squared *p*-value for retro-orbital pain and fever were < 0.01 and = 0.02, respectively). All subjects positive to ZIKV presented exanthema, pruritus, myalgia and prostration. Among the study participants, two and one participants from ZIKV and control groups, respectively, were positive when submitted to the RT-qPCR for detection of dengue types 1 to 4.

**Table 1:** Clinical and demographic characteristics of ZIKV and control patients.

Characteristics	Controls (*n* = 12)	ZIKV patients (*n* = 12)	*P-*value
Age in years (mean ± SD)	43 ± 29	42 ± 18	0.47
Male gender (%)	1 (8)	1 (8)	0.76
Positive for Dengue (RT-PCR) (%)	1 (8)	2 (17)	0.54
History of hypertension (%)	1 (8)	2 (17)	0.54
Fever (%)	6 (50)	11 (92)	0.02
Arthralgia (%)	10 (83)	11 (92)	0.54
Headache (%)	9 (75)	10 (83)	0.61
Exanthema (%)	11 (92)	12 (100)	0.31
Pruritus (%)	10 (83)	12 (100)	0.14
Myalgia (%)	9 (75)	12 (100)	0.06
Tingling (%)	3 (25)	7 (58)	0.1
Dormancy (%)	3 (25)	7 (58)	0.1
Retro-orbital pain (%)	2 (17)	11 (92)	<0.01
Edema (%)	8 (67)	8 (67)	1.0
Lymphadenopathy (%)	1 (8)	5 (42)	0.06
Prostration (%)	9 (75)	12 (100)	0.06


*Statistical analyses were performed using *t* tests for means and chi-square tests for proportions.*

### General Description of Lipidomics Data

**FIGURE 1:**
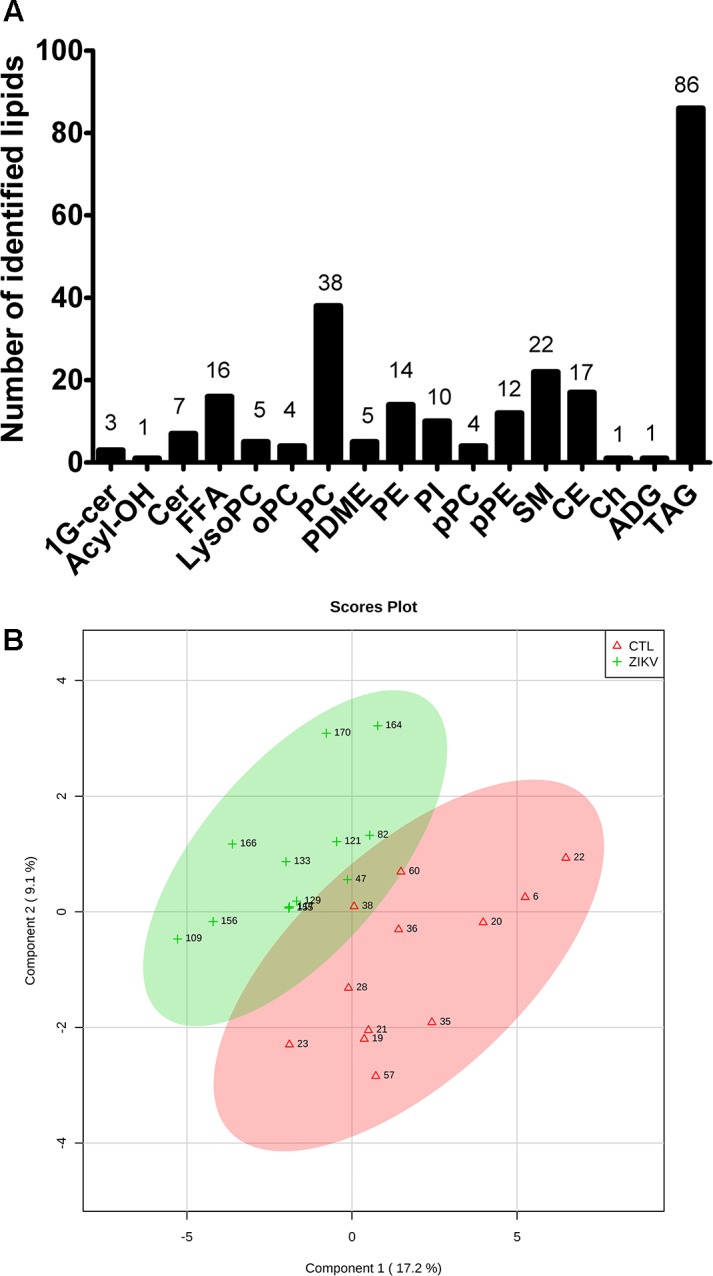
Global lipidomics analysis of serum of ZIKV patients. Number of identified lipid species in ZIKV and control groups by untargeted LC-MS analysis **(A)** and sparse partial least squares discriminant analysis (sPLS-DA) of ZIKV infection (ZKV) and controls (CTL) **(B)**. 1G-cer, Glycosylceramide; Acyl-OH, Hydroxy-acyl; Cer, Ceramide; FFA, Free fatty acid; LysoPC, Lyso-phosphatidylcholine; oPC, Plasmenyl-phosphatidylcholine; PC, Phosphatidylcholine; PDME, Dimethylethanolamine; PE, Phosphatidylethanolamine; PI, Phosphatidylinositol; pPC, Plasmanyl-phosphatidylcholine; pPE, Plasmenyl-phosphatidylethanolamine; SM, Sphingomyelin; CE, Cholesteryl ester; Ch, Cholesterol; ADG, Alkyldiacylglycerol and TAG, Triacylglycerol.

Untargeted lipidomic analysis by liquid chromatography coupled to high-resolution mass spectrometry (LC-MS) was performed in both negative and positive electrospray ionization modes. Manual annotation of lipids based on their MS/MS spectra enabled us to unambiguously identify 246 lipid molecular species distributed into free fatty acids, glycerophospholipids, sphingolipids, triglycerides, cholesterol and cholesteryl esters (Figure 1A). These lipids were quantified using class-specific internal standards (Supplementary Table S1).

### Statistical Analyses

**FIGURE 2:**
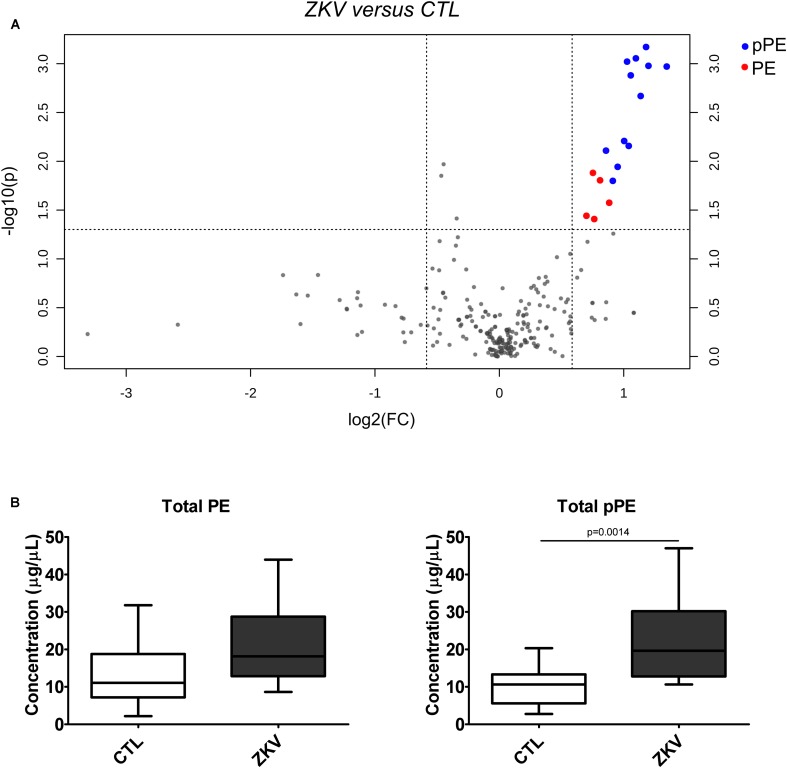
Volcano plot **(A)** of the lipid molecular species altered in ZIKV patients vs. control group. The log_2_ fold change (FC) of ZIKV vs. CTL group was plotted against the -log_10_ *p*-value. Statistical significance was evaluated by *t*-test (*p*-value < 0.05) and FC threshold was set to higher than 1.5. Dots in red and blue represent significantly altered pPE and PE species, respectively. Total PE and pPE levels in ZIKV-infected patients **(B)**. Data are expressed as mean ± standard error (*n* = 12 for each group). Comparison between the groups was performed using *t*-test. A *p*-value < 0.05 was considered statistically significant. Total PE and pPE levels were calculated as the sum of all lipid species in each of the lipid classes. Concentration values are given in μg lipid/μL of serum.

**FIGURE 3:**
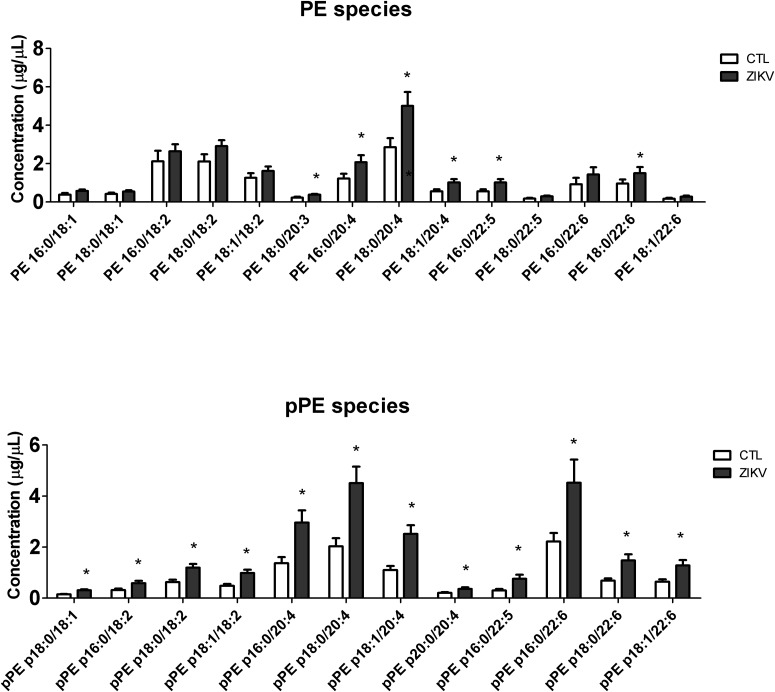
Serum concentration of molecular species of PE and pPE in ZIKV infected (ZKV) and controls (CTL) patients. Concentration values are given in μg lipid/μL of serum and data represented as mean ± standard error (*n* = 12 for each group). Comparison between the groups was performed using *t*-test (*p*-value < 0.05) and significantly altered species are marked with ^∗^. Identified lipid species were annotated by a shorthand nomenclature corresponding to the level of detail attainable by the analysis (e.g., PE 16:0/20:4). The symbol “/” denotes constituting fatty acids moieties of the lipid species, and not their sn-1 and sn-2 position on the glycerol-backbone.

Partial least squares discriminant analysis was performed with the lipidomics data obtained by LC-MS, revealing a clear separation between the ZIKV-infected individuals and controls (Figure 1B). We next constructed a volcano plot applying a significance level of *p-*value < 0.05 based on *t*-test and a FC value higher than 1.5 (Figure 2A). The volcano plot analysis revealed 18 lipid molecular species significantly increased in ZIKV-infected patients relative to the control group (Table 2). From these altered lipids, six species belonged to the subclass phosphatidylethanolamine (PE) and the other 12 were represented by surprisingly all plasmenyl-phosphatidylethanolamine (pPE) or plasmalogens identified in this study (Figure 3). As shown in Figure 2B, there were no significant differences between ZIKV and control groups in respect to total serum concentrations of PE, whereas total plasmalogens were significantly increased in ZIKV patients. Furthermore, the great majority of these altered phospholipids were linked to at least one chain of polyunsaturated fatty acids (fatty acid chains with two or more double bonds, Figure 3). Taken together, our findings highlight the importance of PE, mainly plasmalogens species linked to polyunsaturated fatty acids, as potential serum biomarkers of the ZIKV infection.

**Table 2:** Lipid molecular species altered in serum of ZIKV *versus* control patients as revealed by volcano plot analysis.

Lipid species^∗^	FC	log2(FC)	raw.pval	-log10(p)
pPE p18:0/20:4	2.265	1.180	0.001	3.171
pPE p18:0/22:6	2.140	1.098	0.001	3.055
pPE p18:0/18:1	2.037	1.027	0.001	3.021
pPE p18:1/20:4	2.295	1.199	0.001	2.978
pPE p16:0/22:5	2.541	1.346	0.001	2.971
pPE p18:1/18:2	2.080	1.056	0.001	2.880
pPE p16:0/20:4	2.198	1.136	0.002	2.669
pPE p18:1/22:6	2.005	1.004	0.006	2.208
pPE p16:0/22:6	2.057	1.040	0.007	2.157
pPE p20:0/20:4	1.812	0.857	0.008	2.109
pPE p18:0/18:2	1.933	0.951	0.011	1.943
PE 18:0/22:5	1.685	0.752	0.013	1.881
PE 18:0/20:4	1.753	0.810	0.016	1.805
pPE p16:0/18:2	1.882	0.912	0.016	1.800
PE 16:0/22:5	1.844	0.883	0.027	1.575
PE 18:1/20:4	1.844	0.883	0.027	1.575
PE 18:0/20:3	1.625	0.700	0.036	1.441
PE 16:0/20:4	1.697	0.763	0.039	1.408


*^∗^Annotation of lipids: headgroup abbreviation followed by side-chain characteristics. For x:y number of carbons (*x*) followed by number of double bonds (*y*). For plasmalogens px:y indicates the number of carbons and double bonds in the ether-linked vinyl chain, followed by the fatty acid chain characteristics. Identified lipid species were annotated by a shorthand nomenclature corresponding to the level of detail attainable by the analysis (e.g., PE 16:0/20:4). The symbol “/” denotes constituting fatty acids moieties of the lipid species, and not their sn-1 and sn-2 position on the glycerol-backbone.*

### Receiver Operating Characteristic Curve Analysis

**FIGURE 4:**
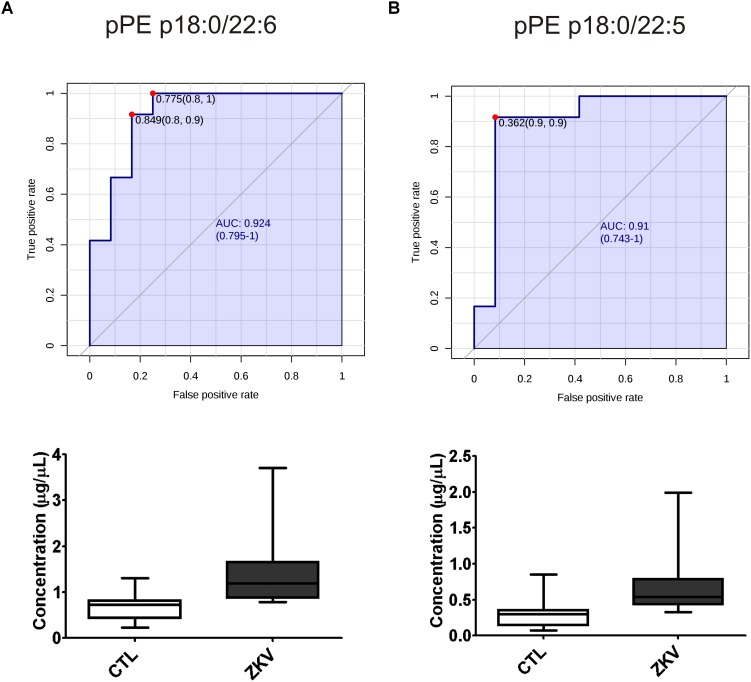
Receiver operating characteristic (ROC) curves and serum concentration of the top 2 pPE lipid molecular species significantly increased in ZIKV-infected patients. pPE p18:0/22:6 **(A)** pPE p16:0/22:5 **(B)** using AUC > 0.9 with a 95% interval confidence for comparing ZIKV patients and control groups. The ROC curve analysis of potential biomarkers was calculated with Youden’s index. ROC analysis was performed without log_10_ transformation.

The fact that 9 out of 18 altered lipids were increased by 2 to 2.3 folds in the serum of ZIKV patients relative to controls prompted the evaluation of these lipids as possible biomarkers for ZIKA infection. For this purpose, we carried out a ROC curve analysis as a diagnostic test and calculated the AUC that expresses the accuracy of the test. As exemplified in Figure 4, pPE (p18:0/22:6) and pPE (p16:0/22:5) displayed increased concentrations in the serum of ZIKV patients relative to controls (*p*-value < 0.001) and AUC values greater than 0.9. In total, our data revealed 12 lipid species that displayed AUC values higher than 0.8, 11 of these are pPE and one cholesteryl ester linked to a 14:0 fatty acid chain (Supplementary Table S2), suggesting over 80% chance of these molecules correctly discriminate a ZIKV infected patient from the control group.

## Discussion

Our strategy in this study was to compare the serum lipid profile between ZIKV patients and symptomatic controls that presented virus-like symptoms as fever, arthralgia, headache, exanthema, myalgia and pruritus. Retro-orbital pain and fever were the only clinical symptoms that significantly distinguished ZIKV and control groups (Table 1). A recent study by Melo et al. (2017) showed that the differences in metabolic and lipid profiles in serum of ZIKV patients are so pronounced that they can be differentiated from a control group composed of both healthy and symptomatic subjects. Thus, by using symptomatic patients as control, we assume that lipid alterations are largely related to ZIKV rather than any other viruses, and likely accounts for the heterogeneity between symptomatic and healthy subjects.

Alterations in lipid homeostasis have been associated with many viral infections (Meertens et al., 2012; Martin-Acebes et al., 2014; Richard et al., 2015; Carnec et al., 2016; Jordan and Randall, 2016). While most of the information about lipid metabolism of host cells and flavivirus come from *in vitro* experiments, there exist just a few, but relevant studies evidencing alterations in serum lipidome caused by flavivirus infection (Cui et al., 2013; Schoeman et al., 2016; Melo et al., 2017, 2018). Fortunately, one of these studies was performed with ZIKV using a metabolomics approach with direct infusion mass spectrometry and database search for ion characterization (Melo et al., 2017). The authors identified four phosphatidylinositol phosphates and one ganglioside as potential biomarkers of the serum lipidome in ZIKV infected subjects. Here, we provide complementary results by applying an untargeted LC-MS lipidomics approach, in which the identification is exclusively based on MS/MS spectra and internal standards are used for semi-quantification of the most abundant lipids. Our results reveal increased levels of six PE and all twelve detected pPE or plasmalogens in serum of ZIKV patients relative to controls (Figure 2, 3). Increased levels of plasmalogens have been also detected in the serum of chronic HBV patients (Schoeman et al., 2016) as well as a strong enrichment of plasmalogens was noticed in the virion lipidome of human cytomegalovirus (Liu et al., 2011) and HIV (Brugger et al., 2006). Interestingly, among the altered phospholipid species found in our study, plasmalogens displayed the highest FC and AUC values (Table 2 and Supplementary Table S2) strongly implicating their elevated concentrations in ZIKV patients as potential diagnostic markers for the disease.

Plasmalogens are synthesized by peroxisomes and represent a unique class of ether-linked glycerophospholipids that contain a vinyl ether bond at the sn-1 position of the glycerol moiety (Fahy et al., 2005; Braverman and Moser, 2012). Virus-induced alterations in both plasmalogens levels and peroxisome activity have been described at the host cell level (Satoh et al., 1990; Farooqui and Horrocks, 2001; Dixit et al., 2010; Odendall and Kagan, 2013; Martin-Acebes et al., 2014). In human hepatocarcinomas, extremely high levels of both pPE and plasmenyl-phosphatidylcholine (pPC), accounting for > 60% of total glycerophospholipids, were observed in cell lines persistently infected by HBV in comparison with < 12% in non-HBV-related hepatocellular carcinomas (Satoh et al., 1990). West Nile virus (WNV) infection alters the lipid metabolism of infected host cells, resulting in increased levels of pPE and pPC by 1.6 and 1.9 FC, respectively, compared to control cells (Martin-Acebes et al., 2014). It has been suggested that these flaviviruses use plasmalogens as preferred components to build their envelope. Recently, pPC was also shown to play a crucial role in influenza virus infection (Tanner et al., 2014). These authors demonstrated that either pharmacological interference of peroxisomal fatty acid β-oxidation or genetic ablation of ether lipid biosynthesis *in vitro* impaired influenza virus replication.

Indeed, there is a growing number of studies suggesting that peroxisomes are key players in antiviral signaling (Dixit et al., 2010; Odendall and Kagan, 2013). For instance, You et al. (2015) have shown that flaviviruses infection, such as by WNV and dengue virus (DENV), results in significant loss of peroxisomes in mammalian cells by capsid protein-dependent sequestration and/or degradation of the peroxisomal biogenesis factor Pex19 (You et al., 2015). More recently, it has been found that NS2A, a ZIKV protein, is targeted specifically to peroxisomal membranes (Coyaud et al., 2018). Consistently, these authors used skin fibroblast cell lines to demonstrate that functional peroxisomes are required for efficient ZIKV replication. The observation that flaviviruses induce peroxisome mediated lipid alterations in infected cells may explain the up-regulation of plasmalogen levels in serum lipidome of ZIKV-infected patients (Figure 3).

Plasmalogens are major constituents of neural membranes and are involved in many neural lipid-mediated processes such as membrane fusion, ion transport, reservoir for second messengers, cholesterol efflux and as antioxidants (Farooqui and Horrocks, 2001). Likewise, peroxisomal metabolism is crucial for proper brain development and function, and it is well known that pathological aberrations of the nervous system are prominent features in most peroxisomal disorders (Berger et al., 2016). To date, there exists no evidence for an increase in plasmalogens concentration in serum of ZIKV infected subjects linked to neural membrane disturbance, nor for interaction of ZIKV with peroxisomes of neural cells. Since ZIKV has been involved in neonatal microcephaly and Guillain–Barré syndrome in adults (White et al., 2016), we can only speculate that the infection with ZIKV causes alterations in the nervous system. Nonetheless, it is known that plasmalogens are broadly distributed over brain, heart, kidney, skeletal muscle (Braverman and Moser, 2012). Without further experiments, we are unable to pinpoint which of these tissues might represent a potential source of plasmalogens detected in serum of ZIKV infected patients.

Cholesterol, PS, and PE have been suggested as major lipids of flavivirus envelopes (Meertens et al., 2012; Richard et al., 2015; Martin-Acebes et al., 2016; Wewer and Khandelia, 2018). In addition, both PE and PS associated with virus particles are important sites for binding host cell receptors in DENV attachment and internalization (Perera-Lecoin et al., 2013; Carnec et al., 2016) as well as TIM and TAM family members in permissive skin cells, during ZIKV infection (Hamel et al., 2015). PE is also associated with Ebola, DENV and WNV and plays a key role in TIM1-mediated virus entry (Richard et al., 2015). Interesting, TIM1 is a cofactor universally expressed across placental cells and binds to PE during ZIKV infection (Tabata et al., 2016). Although we have not observed any alteration in PS and cholesterol concentrations, the level of several PE and plasmalogens species was significantly increased in ZIKV patients relative to controls (Table 2 and Figure 3).

Lipids are of central importance for enveloped viruses replication since they affect the generation and maintenance of cellular membrane curvature (Martin-Acebes et al., 2014), thereby affecting the virus entry into host cells by fusion of viral membranes with cellular membranes. As mentioned above, up-regulated plasmalogens were mostly esterified to either omega-3 or -6 polyunsaturated fatty acids such as oleic, arachidonic, docosapentaenoic and docosahexaenoic (DHA) acids (Table 2). Plasmalogens have been reported to possess very distinct physical properties than their diacyl counterparts (i.e., PE), in that they are considered highly fusogenic lipids (Glaser and Gross, 1995) and tend to form a more densely packed and thicker bilayer (Rog and Koivuniemi, 2016). The increased abundance of plasmalogens attached to polyunsaturated fatty acids in the serum of ZIKV patients may have important implications for viral vesicles fusion with cellular membranes as well as for their stability in the extracellular space. Moreover, polyunsaturated fatty acids linked to glycerophospholipids are substrates for the generation of soluble lipid mediators that participate in cell signaling either by inducing or resolving inflammation. For instance, several lipid mediators derived from the enzymatic oxidation of oleic acid and DHA have been recently implicated in immune status (Tam et al., 2013) and antiviral response (Morita et al., 2013) in mouse models of influenza virus infection.

Members of *Flaviviridae* family such as DENV, WNV and also ZIKV are enveloped viruses that hijack host cell lipids to build their particle envelopes. The alteration in lipid homeostasis of host cells is a viral strategy to create a proper environment for replication. Our results indicate that the concentrations of several lipids molecular species of PE (both PE and plasmalogens) linked to polyunsaturated fatty acids were increased in the serum of ZIKV infected patients compared to controls. Of note, the association of all lipid molecular species of plasmalogens with ZIKV patients supports recent data evidencing the importance of these lipids for viral lifecycle (Tanner et al., 2014; Coyaud et al., 2018) and thus their potential application as diagnostic/prognostic markers. Our findings provide many insights for follow-up studies using host cells, in particular approaches to investigate lipid metabolic pathways, and their potential use as therapeutic targets for ZIKV infection.

## Ethics Statement

The institutional review boards at the Instituto Gonçalo Moniz (Fiocruz-Bahia) approved the present study (CAAE: 1.100.349) and the subject provided written and informed consent prior to participation.

## Author Contributions

AQ, AF, VA, MY, SM, and LA designed research. AQ, MY, ML, MG, JJ, JX, FB, IP, HA, AF, DM, MF, NS performed the research. AQ, IP, MY, and GC analyzed data. AQ, IP, VA, MY, SM, and LA wrote the manuscript.

## Conflict of Interest Statement

The authors declare that the research was conducted in the absence of any commercial or financial relationships that could be construed as a potential conflict of interest.
