# Supplementary material for: Lipidomic Analysis Reveals Serum Alteration of Plasmalogens in Patients Infected With ZIKA Virus
Source: Front Microbiol. 2019 Apr 12;10:753. doi: 10.3389/fmicb.2019.00753 (PMC6474330; doi:10.3389/fmicb.2019.00753)
Supplement: Supplementary file 1 [file Table_1.docx]

***Supplementary Material***

**Supplementary table 1**. Lipid subclasses and their respective internal standards applied in the untargeted lipidomics analysis.

| **Class** | **Subclass** | **Abbreviation** | **Standard^¥^** |
| --- | --- | --- | --- |
| Fatty acids | Free fatty acid | FFA* | PC (17:0/17:0) |
| Glycerophospholipids | Phosphatidylethanolamine | PE | PE (17:0/17:0) |
|  | Plasmenyl-phosphatidylethanolamine | pPE | PE (17:0/17:0) |
|  | Phosphatidylcholine | PC | PC (17:0/17:0) |
|  | Lyso-Phosphatidylcholine | LysoPC | LPC (17:0) |
|  | Plasmanyl-phosphatidylcholine | oPC | PC (17:0/17:0) |
|  | Plasmenyl-phosphatidylcholine | pPC | PC (17:0/17:0) |
|  | Phosphatidyl-dimethylethanolamine | PDME | PC (17:0/17:0) |
|  | Phosphatidylinositol | PI* | PC (17:0/17:0) |
| Sphingolipids | Sphingomyelin | SM | SM (d18:1/17:0) |
|  | Ceramide | Cer | Cer (d18:1/17:0) |
|  | Glucosylceramide | 1G-Cer | Cer (d18:1/17:0) |
| Sterols | Cholesterol | Ch* | PC (17:0/17:0) |
| Storage  Lipids | Cholesteryl ester | CE* | PC (17:0/17:0) |
|  | Triacylglycerol | TAG | TAG (14:0/14:0/14:0) |
|  | Alkyldiacylglycerol | ADG | TAG (14:0/14:0/14:0) |

¥ Annotation of lipids: headgroup abbreviation followed by side-chain characteristics. For glycerolipids x:y number of carbons (x) followed by number of double bonds (y). For sphingolipids dx:y indicates the number of carbons and double bonds in the sphingosine backbone, followed by the n-acyl chain characteristics. *these lipid subclasses were normalized by PC (17:0/17:0) using correction factors performed with external calibration curves with the respective external standards. The following correction factors were applied to these subclasses: 2.94 (FFA); 0.65 (PI), 0.17 (Ch) and 0.0098 (CE).
