# Supplementary material for: Lipidomic Analysis Reveals Serum Alteration of Plasmalogens in Patients Infected With ZIKA Virus
Source: Front Microbiol. 2019 Apr 12;10:753. doi: 10.3389/fmicb.2019.00753 (PMC6474330; doi:10.3389/fmicb.2019.00753)
Supplement: Supplementary file 2 [file Table_2.docx]

***Supplementary Material***

**Supplementary table 2.** The AUC, 95% confidence intervals (95% CI), and sensitivity and specificity for ROC curves

| **No** | **Lipid species** | **AUC** | **95%CI** | **Sens.,Spec.** | **Cutoff value** | **P value** | **Log2 FC** |
| --- | --- | --- | --- | --- | --- | --- | --- |
| 1 | pPE p18:0/22:6 | 0.924 | 0.795-1 | 0.8, 0.9 | 0.849 | 0.005 | -1.0893 |
| 2 | pPE p16:0/22:5 | 0.91 | 0.743-1 | 0.9, 0.9 | 0.362 | 0.011 | -1.3575 |
| 3 | pPE p18:0/20:4 | 0.889 | 0.729- 0.976 | 0.7, 1.0 | 2.24 | 0.001 | -1.2642 |
| 4 | pPE p18:1/18:2 | 0.889 | 0.705-0.979 | 0.9, 0.8 | 0.697 | 0.001 | -1.1644 |
| 5 | pPE p18:0/18:1 | 0.875 | 0.712-1 | 0.9, 0.8 | 0.215 | 0.000 | -1.059 |
| 6 | pPE p18:1/20:4 | 0.882 | 0.708-0.979 | 0.9, 0.8 | 1.62 | 0.001 | -1.2915 |
| 7 | pPE p16:0/20:4 | 0.854 | 0.674-0.972 | 0.8, 0.8 | 1.6 | 0.005 | -1.214 |
| 8 | CE 14:0 | 0.840 | 0.635-0.979 | 1.0, 0.7 | 7.12 | 0.041 | 0.65767 |
| 9 | pPE p16:0/22:6 | 0.826 | 0.628-0.965 | 0.6, 1.0 | 2.22 | 0.003 | -1.1603 |
| 10 | pPE p18:0/18:2 | 0.819 | 0.594-0.948 | 0.9, 0.6 | 1.07 | 0.003 | -1.1603 |
| 11 | pPE p18:1/22:6 | 0.812 | 0.614-0.944 | 0.8, 0.7 | 0.898 | 0.010 | -1.0702 |
| 12 | pPE p20:0/20:4 | 0.806 | 0.604-0.979 | 0.8, 0.8 | 0.299 | 0.009 | 0.507 |

Sens = sensibility; Spec = specificity; FC = fold change. ROC curve analysis without log10 transformation.
